# Supplementary material for: Are carbon emissions trading and green financial instruments synergistic? -Comprehensive quantitative research based on content analysis
Source: PLoS One. 2024 Mar 7;19(3):e0298601. doi: 10.1371/journal.pone.0298601 (PMC10919601; doi:10.1371/journal.pone.0298601)
Supplement: S1 Data — (ZIP) [file pone.0298601.s001.zip › Data/地级市Z_F工作报告.zip!/╣π╢1⁄2/╜¡├┼╩╨/└┤╫╘╣1⁄2╓┌║┼í╛┬φ┐╦╩2╛▌═°í┐_2011.docx]

各位代表：

　　我代表市人民政府向大会作政府工作报告，请予审议，并请政协各位委员和其他列席人员提出意见。

　　“十一五”时期的工作回顾

　　2010年主要工作回顾

　　预计2010年全市生产总值突破1500亿元（达1550亿元），比上年增长14.3%:

　　地方财政一般预算收入突破100亿元（达104.29亿元），比上年增长24.7%;

　　规模以上工业增加值突破800亿元（达829.78亿元），比上年增长23.7%;

　　全社会固定资产投资突破600亿元（达631.77亿元），比上年增长28.4%;

　　社会消费品零售总额突破600亿元（达655.86亿元），比上年增长17%;

　　金融机构本外币各项贷款余额突破1000亿元（达1032.46亿元），比年初增长15.67%;

　　外贸进出口总额达140亿美元，比上年增长27%;

　　实际利用外商直接投资11.06亿美元，比上年增长6.7%;

　　“十一五”时期是我市改革开放和现代化建设取得新的重大成就的五年。市政府在省委、省政府和市委的正确领导下，在市人大及其常委会和市政协的监督支持下，深入贯彻落实科学发展观，以实施《珠三角规划纲要》为动力，有效应对国际金融危机，扎实推进“三促进一保持”，全面完成了“十一五”规划和2010年各项预期目标任务。

　　——经济综合实力跨上历史性新台阶。

　　预计2010年全市生产总值突破1500亿元（达1550亿元），比上年增长14.3%。地方财政一般预算收入突破100亿元（达104.29亿元），比上年增长24.7%。规模以上工业增加值突破800亿元（达829.78亿元），比上年增长23.7%。全社会固定资产投资突破600亿元（达631.77亿元），比上年增长28.4%。社会消费品零售总额突破600亿元（达655.86亿元），比上年增长17%。金融机构本外币各项贷款余额突破1000亿元（达1032.46亿元），比年初增长15.67%。外贸进出口总额达140亿美元，比上年增长27%。实际利用外商直接投资11.06亿美元，比上年增长6.7%。

　　经过五年的努力，全市经济综合实力显著增强，生产总值五年年均增长13%，提前一年实现“十一五”发展目标。三大产业结构由“十五”期末的9:52.8:38.2演变为7.5:56.5:36。地方财政一般预算收入、工业增加值、固定资产投资、社会消费品零售总额和金融机构各项贷款余额等主要经济指标实现五年翻一番。地方财政一般预算收入年均增长20.2%，比“十五”期末增加62.66亿元。规模以上工业增加值年均增长18.9%，比“十五”期末增加474.68亿元。固定资产投资年均增长21.6%，五年累计固定资产投资超2000亿元，超过改革开放以来至2005年27年的投资总和。社会消费品零售总额年均增长16.2%，比“十五”期末增加346.04亿元。金融机构本外币各项贷款余额年均增长14.94%，比“十五”期末增加568.97亿元。五年来累计实际利用外商直接投资45.21亿美元，其中超千万美元项目209个。

　　——现代产业体系建设取得新成效。

　　大项目引进和战略性新兴产业培育取得重大突破，台山核电、广东轨道交通车辆修造基地、省清洁能源（核电）装备产业园、富华重工、高新区LED产业基地和博览交易中心、银洲湖纸业基地等项目建设进展顺利。19个项目列入省现代产业“500强”。自主创新能力显著提升，五年新增34家国家级高新技术企业、98个省市级工程研发中心、5个中国名牌产品和12个中国驰名商标，累计分别达84家、150个、14个和13个。成功创建摩托车国家检测中心，获批成立国家半导体光电产品检测重点实验室、国家机械装备检测重点实验室和3个省级检测站。4个项目列入省自主创新“100强”。先进制造业、现代农业和现代服务业加快发展。五年新增6个国家级产业基地，累计达16个。江门高新区晋升为国家级高新技术开发区。江门产业转移工业园被认定为省示范性产业转移工业园。成功创建3个国家级循环经济试点和2个省级循环经济试点单位。海洋经济进一步壮大，产值达270亿元，比“十五”期末增长294.56%。农业产业化进程加快，全市市级以上重点农业龙头企业、农民专业合作经济组织比“十五”期末增加34家和119家，分别达57家和162家，带动全市52%的农户户均增收2160元。创建了全省首个国家现代农业示范区和省粤台农业试验区。旅游业实现总收入119.7亿元、外汇收入31.62亿元，分别比“十五”期末增长140.85%和263.45%。现代物流业实现增加值210亿元，比“十五”期末增长96.63%。金融对经济发展的支撑力显著增强，引进了交通银行、汇丰银行、民生银行、中信银行、兴业银行、顺德农商银行以及4家小额贷款公司开办分支机构，农信社改革工作扎实推进，新增1家境内上市公司。预计节能减排完成省下达任务，全市化学需氧量、二氧化硫排放量分别比“十五”期末下降9.1%和18.2%。土地管理和节约集约用地得到新加强。

　　——落实《珠三角规划纲要》有新突破。

　　坚持“左接广佛，右联珠中”，与广佛、珠中及港澳合作全面展开。珠中江城市（镇）供水同网实施方案正式启动，三市跨市公交线路开通，在全省率先实现珠中江年票互认，潮连大桥收费站对佛山车辆实行免费通行。实施西江水环境共治，完成市区21家污染企业搬迁。主动承接广佛都市圈辐射，富华重工成为全省最大“双转移”项目，开平市和顺德区达成了合作建设翠山湖产业转移园的协议。市区一体化稳步推进，实现市域年票制、社会保障“一卡通”、公交“一卡通”和出租车三区通行，完成养老、医疗、失业、工伤、生育保险和城乡居民医保市级统筹。

　　——宜居城乡建设实现新跨越。

　　城乡交通基础设施建设取得重大进展，成为179个国家公路运输枢纽之一。滨江大道、广珠城轨江门支线、江珠高速、江肇高速建成通车。建成篁庄大道、胜利北路、文昌沙水质净化厂等重点城市建设项目，东华大桥、胜利大桥动工建设。滨江新区建设全面铺开，新区道路交通、水利等基础设施规划建设加快推进。“三旧”改造扎实推进。全市286公里省立绿道全线贯通。农村生产生活条件明显改善，村庄整治任务完成率达40%，全市11.5万人的农村饮用水不安全问题得到全面解决。生态环境保护成效明显，已建成生活污水处理设施26座、截污管网198公里，日处理能力49.3万吨，城镇生活污水处理率达到60%以上，工业废水排放达标率达到90%以上，工业固体废物处置利用率达到95%以上。全市大气环境质量维持在国家二级标准，市区空气质量日报优良率98%，城市水域功能区和饮用水源水质达标率100%。建成区绿化覆盖率达40.75%，人均公园绿地面积达11平方米。

　　——改革开放迈出新步伐。

　　完成市、县两级政府机构改革，市政府工作部门由36个减到32个。成为省创新审批方式改革试点，85%以上的审批事项已下放到各市、区。“大部制”试点、富县强镇、简政强镇事权改革稳步推进。基本完成林改主体改革任务。对外开放迈上新水平，成功承办粤台经贸交流会和珠中江进出口商品展销会，获“中国摩托车出口基地”和“中国麦克风出口基地”称号。民营经济加快发展，全市民营企业户数和注册资金分别达2.39万户、321.83亿元，比“十五”期末增长66.89%和1.88倍。国企改革深入推进，市直国有企业实施改制45户，安置职工1.3万人，退休人员移交社区管理1.5万人，市直国有企业总体实现扭亏为盈。

　　——人民生活水平有新提高。

　　各级财政民生投入不断加大，五年累计投入190.58亿元，年均增长28.19%。城镇居民人均可支配收入、农村居民人均纯收入分别达21153元和8607元，分别比上年增长11.3%和14.2%，比“十五”期末增长63.9%和60.9%。城乡居民本外币储蓄存款余额1516.72亿元，比“十五”期末增加564.76亿元。不断健全社会保障体系，全市城镇职工基本养老、基本医疗、失业、工伤、生育保险参保人数分别比“十五”期末增长37%、172%、45%、56%、81%。城镇居民基本医疗保险实现全覆盖，新型农村合作医疗参合率由“十五”期末的71.27%提高到99.73%，保障水平稳步提高。新型农村社会养老保险试点扎实推进。就业和再就业工作取得新成绩，城镇登记失业率控制在2.49%以内，低于全省平均水平。“十一五”期间全市新增城镇就业岗位20.73万个，安置城镇人员就业20.04万人。实施“千企扶千村”就业工程，免费培训农村富余劳动力20.43万人，培训后转移就业17万人，就业率为83%。高度重视解决低收入群体生活问题，五年共支出低保资金3.2亿元，救助低保对象3.05万户、7.45万人。大力推进住房保障工作，廉租住房和经济适用住房保障家庭分别新增1480户和433户。慈善事业蓬勃发展，接收捐款共计4.5亿元，投放善款逾3亿元。大力发展义工事业。“平安江门”建设稳步推进，刑事案件发案数呈逐年下降趋势。

　　——文化名市建设有新飞跃。

　　创建全国文明城市取得阶段性成效，成为“全国创建文明城市工作先进城市”和全国创建文明城市先进典型。开平碉楼与村落成为全省首个世界文化遗产项目。华人嘉年华暨侨乡旅游节和侨乡书香节品牌进一步打响。新建成五邑华侨华人博物馆、江门美术馆、星光公园、市青少年宫、五邑文化广场、市广播电视中心等一批公共设施。大力推进农村公共文化建设，基本实现了95%以上的社区、80%以上的村建有公共文化场所和设施，广播电视实现了“村村通”。文化产业迅速发展，形成了印刷、文化旅游、动漫等三大主导产业。

　　——各项社会事业有新进展。

　　成功创建广东省教育强市。全面实现城乡免费义务教育，实施义务教育学校绩效工资，落实中小学教师工资福利待遇“两相当”，全面解决代课教师问题。城乡医疗卫生服务体系进一步健全，中医药名市建设深入推进，健康城市建设初见成效，“十一五”期间成功创建国家、省、市卫生镇15个和卫生村308条。体育事业取得历史性突破，我市体育健儿在北京奥运会和残奥会以及广州亚残运会上勇夺三金两银一铜，全民健身活动蓬勃开展。连续五年完成省下达的人口计生任务，市区及开平市、鹤山市全面实现免费婚检和孕前检查。残疾人工作上新水平。侨务、外事、港澳、对台、民族、宗教、老龄、统计、审计、新闻、出版、档案、方志、气象、红十字会等工作均取得新进步。

　　——服务型政府建设得到新加强。

　　推动机关作风改进和效率提高的一系列措施取得实效，“12345”政府服务热线覆盖全市，江门市新行政服务中心和新会区、开平市、台山市的行政服务中心建成并投入使用。自觉接受市人大及其常委会和市政协的监督，创新推行人大代表约见市长、局长制度，认真办理人大议案、代表建议和政协提案，“十一五”期间共办理人大代表议案3件、代表建议519件，满意率为97.9%；办理政协提案656件，满意率为99.85%。建立信访维稳“一岗双责”责任制，构建市、县、镇、村四级信访工作网络，加快综治信访维稳中心建设，有效解决一大批涉及群众切身利益的问题。干部法律意识不断提高，效能监察、廉政建设和反腐败工作进一步加强，查处一批违纪违法案件。打私、监狱、劳教工作得到加强。产品质量和食品药品安全监管整治取得实效。荣获“全国双拥模范城”四连冠、“广东省双拥模范城”六连冠和“全国人民防空先进城市”称号。安全生产隐患治理成效显著，重特大事故得到有效遏制。完善应急预案体系，应急处置能力得到提高。举全市之力支援灾区抗震救灾和对口援建，以全省第二的速度提前完成省下达我市的援建任务。“双到”工作取得阶段性成果，落实“双到”帮扶资金9618万元，启动帮扶项目946个，省下达任务的105条贫困村集体收入全部达3万元以上，6166户、占总数68%的贫困户实现脱贫。

　　上述成绩的取得，是在省委、省政府和市委的正确领导下，全市人民团结一致、开拓创新、奋力拼搏的结果。在此，我代表市人民政府，向全市广大工人、农民、知识分子、干部职工和外来建设者，向驻我市人民解放军、武警官兵、人民警察和各民主党派、人民团体、社会各界人士，致以崇高的敬意！向长期关心支持我市发展的港澳台同胞、海外侨胞以及国际友人表示衷心的感谢！

　　回顾五年来的政府工作，通过历届政府的不懈努力，我们积累了许多宝贵经验，为江门科学发展、可持续发展打下了坚实基础。其中，最为突出的有五个方面。

　　一是坚持工业强市，为未来经济腾飞奠定坚实的产业基础。

　　近年来，我们紧紧围绕《珠三角规划纲要》对江门的定位要求，通过制定实施园区带动、龙头拉动、集聚发展、产业招商、企业减负等一系列政策措施，逐步探索出符合江门实际、符合产业规律的发展路子，全市工业规模超4000亿元，形成了以核电新能源、LED新光源、南车新装备、化纤新材料为先导，摩托车、纺织服装、造纸、造船、食品、包装材料、五金卫浴、印刷、机电等为支柱的较为完整、基础雄厚的工业体系，一批工业园区初具规模，一批对产业链发展带动性强的龙头企业纷纷落户，一批上千亿元的产业集群有望形成，迎来了蓄势待发的机遇期。

　　二是坚持走可持续发展道路，为老百姓营造宜居宜业宜游环境。

　　我们始终坚持“既要金山银山，更要绿水青山”的可持续发展理念，发展经济坚决不以牺牲环境、不以浪费资源为代价，严把招商引资投资强度关、产业政策关、税收效益关和环保准入关；城市建设注重以人为本，大力构建“山、水、城、林”融为一体的城市生态环境格局，促进人与自然和谐发展。通过不懈努力，在全市经济保持较快发展的同时，我们保持了“蓝天白云、青山绿水”，全市生态环境优美，交通网络四通八达，老百姓安居乐业，避免了走“先发展、后治理”的弯路。

　　三是坚持加大“三农”工作力度，为实现城乡区域协调发展打下良好基础。

　　我们紧紧围绕我市作为全省农业大市的实际，持续加大财政支农力度，五年全市各级财政“三农”投入140亿元，年均增长33.91%，并坚持多措并举推进“三农”工作，积极推进社会主义新农村建设。“十一五”时期，是我市支农惠农力度最大的五年，是现代农业加快发展、农民收入持续提高、农村和谐稳定的五年，也是三区辐射带动能力提升、四个县级市发展提速的五年，全市城乡区域发展协调性明显增强，为未来经济社会发展起到了强基固本的作用。

　　四是坚持大力改善民生，让人民群众共享改革发展成果。

　　关注民生、保障民生、改善民生是政府一切工作的出发点和落脚点。我们坚持以民生优先为导向，不断加大投入，实施了一系列民生工程，重点解决老百姓关注的社会保障、医疗卫生、教育文化、社会治安、住房保障等问题，全市治安环境良好，社会和谐发展，老百姓满意度比较高，平安江门、和谐侨乡成为城市名片，人民群众的积极性和创造性得到充分发挥，这将成为我们加快发展的不竭动力。

　　五是坚持改进机关作风，努力打造让市民群众满意的政府。

　　近年来，我们坚持面向市民、面向企业、面向社会，打开门进行机关作风评议，建立了一系列改进机关作风的体制机制，“12345，有事找政府”已经成为市民群众的“口头禅”。实践证明，机关作风的不断改进，对密切党和政府与人民群众的关系，对优化投资环境，对提高服务群众、服务投资者、服务企业水平起到了显著的促进作用，而且也必将成为政府建设持之以恒的重要举措。

　　在充分肯定成绩的同时，我们也清醒地看到工作中还存在不少突出问题：在经济发展方面，规模总量仍不够大，经济结构调整步伐仍不够快，产业集聚水平、自主创新能力和产业竞争力不高，服务业在生产总值所占比重偏低，一些基础性、前瞻性、“瓶颈”问题的解决力度还有待进一步加强。在社会民生方面，统筹城乡发展步伐仍不够快，市民对城乡公共交通、医疗卫生服务、市容市貌等还有不满意的地方。在政府服务方面，政府职能转变还没有到位，行政资源有待进一步整合，办事效率离市民、企业的期望还有一定差距。这些问题，我们将高度重视，采取得力措施加以解决。

　　“十二五”时期的奋斗目标和工作思路

　　“十二五”时期是我市加快转变经济发展方式的攻坚时期，是深入实施《珠三角规划纲要》的重要时期，也是我市全面建设更高水平小康社会、向基本实现社会主义现代化目标迈进的关键时期。综观当前形势，我市既面临难得机遇，又面临严峻挑战。改革开放三十年为我市奠定了坚实的发展基础，加上珠三角一体化进程加快，我市地处珠三角的腹地，土地、交通、资源、环境等发展条件优越，必将迎来厚积薄发的机遇期，但我们也面临宏观政策面趋紧，融资、用地、环保等“瓶颈”问题没有得到有效缓解，区域竞争日益激烈的新局面、新挑战。为此，我们必须认清形势，增强加快发展、争先进位的危机感、责任感和使命感，抢抓难得机遇，努力推动全市经济社会发展再上新台阶。市委十一届九次全会通过的《关于制定全市国民经济和社会发展第十二个五年规划的建议》提出的指导思想是：高举中国特色社会主义的伟大旗帜，以邓小平理论和“三个代表”重要思想为指导，深入贯彻落实科学发展观，以科学发展为主题，以加快转变经济发展方式为主线，全面落实《珠三角规划纲要》，大力推进产业结构战略性调整，全力打造珠三角先进制造业重点发展区和生态型宜居典范城市，努力实现“加快转型发展，建设幸福侨乡”的壮丽蓝图，为江门全面建设小康社会，率先基本实现社会主义现代化打下具有决定性意义的基础。

　　“十二五”时期我市经济社会发展的主要目标是：生产总值力争年均增长15%，人均生产总值年均增长14%，到2015年，生产总值、地方财政一般预算收入、全社会固定资产投资、社会消费品零售总额均比2010年翻一番以上，开放型经济水平进一步提高，在经济总量上再造一个江门，在全省排名努力实现争先进位；经济增长的质量效益明显提升，资源利用和环境保护水平显著提高。全力创建全国文明城市，社会各项事业全面发展，人民生活总体达到宽裕小康水平，努力实现“加快转型发展，建设幸福侨乡”的壮丽蓝图。

　　做好“十二五”工作，实现“十二五”目标，必须要明确“一树立、两抓实、三突破、四提升”的工作思路。

　　——“一树立”：就是要牢固树立科学发展是硬道理的理念。未来五年，我市的核心问题还是要加快发展，而且是要科学发展，努力推进人与自然和谐发展、社会与经济协调发展。坚持科学发展，必须要以更加开放的胸怀，站在珠三角一体化和全省科学发展的大局来谋划江门的发展；必须要以更加创新的精神、创造性的思路来破解改革开放和现代化建设道路上的“瓶颈”难题；必须要以求真务实的工作作风，建立起重大项目、重大活动和为群众办实事好事倒逼机制，提高实现目标任务的执行力。

　　——“两抓实”：一是坚持把保障和改善民生作为政府工作的出发点和落脚点来抓实，提高城乡居民收入，制定幸福侨乡的指标体系，并转化成约束性指标，分解到年度考核任务进行督办。二是坚持把“三农”工作作为统筹城乡发展、推进公共服务均等化、促进社会和谐稳定的重点来抓实，加快发展现代农业，提高社会主义新农村建设水平，拓宽农民增收渠道，缩小城乡差别。

　　——“三突破”：就是充分利用江门在“十二五”时期的独特区位优势，把招商引资作为发展“重头戏”，对各市、区进行分类指导，在三方面实现新突破：一是充分利用“左接广佛、右联珠中”的区位优势，主动承接广佛都市圈辐射，在推进“双转移”、建设现代产业体系上有新突破。二是利用全市土地资源丰富、交通网络不断完善的优势，主动承接国内乃至世界经济发展的梯度转移，在对央企、龙头民企、世界500强企业招商上有新突破。三是利用我市海岸线长、港口资源丰富、对外经济联系方便的优势，按照以产业龙头带动大产业链形成的思路，在推进海洋经济大发展上有新突破。

　　——“四提升”：一是提升民营经济和外资经济综合实力和发展水平，把民营经济作为江门未来经济发展的重点来抓，努力培育新经济增长点；推动外经贸从规模速度型向质量效益型转变，鼓励外资企业增资扩产，开拓国内市场。二是提升企业的科技创新水平，加快推进自主创新体系建设，促进企业提升创新能力，推动新兴产业发展和传统产业转型升级。三是提升现代服务业发展规模和水平，把推动服务业大发展作为产业结构优化升级的战略重点，突出发展生产性服务业和旅游业，特别是要充分利用江门生态环境优美、旅游资源丰富的优势，大力整合旅游资源，尽快把旅游业打造成带动服务业大发展新的产业链条和引擎。四是提升中心城市辐射带动能力，加快推进市区一体化，着力提升滨江新区对江门城市化的带动作用和服务功能，加快把滨江新区建设成为现代化、生态城市的核心发展区。

　　各位代表，展望我市“十二五”发展，前景十分广阔。我们坚信，只要全市人民团结一致，共同努力，“十二五”发展的宏伟目标一定能顺利实现！

　　2011年工作安排

　　今年经济社会发展主要预期目标

　　生产总值增长15%;

　　人均生产总值增长14%;

　　全社会固定资产投资增长25%;

　　社会消费品零售总额增长18%;

　　外贸进出口总额增长10%;

　　地方财政一般预算收入增长13%;

　　城镇居民人均可支配收入增长12%，农村居民人均纯收入增长11%;

　　居民消费价格涨幅控制在4%左右;

　　服务业增加值占生产总值比重达38%;

　　研究与发展经费支出占生产总值比重达1.1%左右;

　　城镇登记失业率控制在3.2%以内;

　　单位生产总值能耗下降和二氧化硫、化学需氧量、氨氮、氮氧化物、单位生产总值二氧化碳排放总量减排完成国家、省下达的目标任务;

　　2011年是“十二五”规划的开局之年。今年政府工作的总体要求是以党的十七届五中全会、中央经济工作会议、省委十届八次全会和市委十一届九次全会精神为指导，围绕“加快转型发展，建设幸福侨乡”，深入实施《珠三角规划纲要》，加快转变经济发展方式，坚持扩大内需拓展外需，推进战略性新兴产业发展和现代产业体系建设，加快以滨江新区为重点的生态宜居城乡建设，创建全国文明城市，进一步保障和改善民生，为“十二五”开好局、起好步。全市经济社会发展的主要预期目标是：生产总值增长15%，人均生产总值增长14%，全社会固定资产投资增长25%，社会消费品零售总额增长18%，外贸进出口总额增长10%，地方财政一般预算收入增长13%，城镇居民人均可支配收入增长12%，农村居民人均纯收入增长11%，居民消费价格涨幅控制在4%左右，服务业增加值占生产总值比重达38%，研究与发展经费支出占生产总值比重达1.1%左右，城镇登记失业率控制在3.2%以内，单位生产总值能耗下降和二氧化硫、化学需氧量、氨氮、氮氧化物、单位生产总值二氧化碳排放总量减排完成国家、省下达的目标任务。

　　为实现上述目标，必须做好以下工作：

　　一、加快转变经济发展方式

　　狠抓战略性新兴产业培育壮大。加快现代产业100强项目建设，着力抓好LED、汽车零部件、轨道机车修造、核电装备、新材料等产业链招商和南车、台山清洁能源（核电）装备产业园、富华重工、真明丽、华宸太阳能光伏电池、丰江锂电池等项目建设，扩大战略性新兴产业的投资力度。以推进“双转移”为突破口推动产业发展上新水平，加强对重点地区的产业链招商，特别对广佛都市圈的招商要实现新突破，抓好开平与顺德共建翠山湖产业转移园工作。把招商引资的工作重心放到各市、区，各职能部门要进一步增强服务意识，对项目审批程序简化手续、提高效率。采用聘任制等方式引入高素质的招商引资人才，提高战略性新兴产业特别是世界500强、央企、省企和民营重大项目的招商水平。对引进项目实行全程跟踪服务，抓好李锦记集团、美达锦纶、维达纸业、西铁城集团等增资扩产工作，提高合同资金到位率，力争实际利用外资和制造业利用外资增幅超过全省平均水平。

　　提升工业园区建设水平。加大园区整合力度，加快重点工业园区和产业集聚区建设，提高市国家级高新区的建设和引资水平，力争江门产业转移工业园在全省产业转移工业园评比中争先进位，力争台山工业园区纳入省产业转移园区。找准园区重点发展的主导产业，促进上、中、下游产业良性互动发展，推进产业集聚，力争今年园区招商引资和工业产值增长双双超过30%。找准园区建设和招商引资盈亏平衡点，更加注重环保，更加注重投资强度，更加注重税收效益。大力推进节约集约用地和统筹节约环境容量工作。坚决处置闲置地，整合零散地、低效地，鼓励企业建设高层厂房。新增工业用地项目一律进园区，防止“村村点火、镇镇冒烟”。

　　加快发展现代服务业。充分发挥开平碉楼与村落世界文化遗产的龙头优势，加快新会银湖湾湿地公园游艇休闲度假区、恩平盛林生态旅游度假区、新会希尔顿酒店、鹤山喜来登酒店、江海区海逸酒店等重大旅游项目建设，大力整合全市旅游资源，提升与港澳台、外省及国际旅游合作水平，做大做强旅游业。加快银洲湖物流基地、鹤山物流中心、南新区物流园等园区和绿色光源专业市场、义乌小商品城、正佳商业广场建设，办好首届绿色光源国际博览会、传统家具精品鉴赏会等展会。积极引入银行、证券、保险机构来我市开设分支机构，大力发展小额贷款公司和融资性担保机构，力争年内有1－2家新银行机构开业经营。进一步深化农信社改革，力争2家农信社改制成农村商业银行。努力发展资本市场，力争2－3家企业年内上市。围绕打造中国“动漫侨乡”的战略目标，培育出若干个规模较大、有自主开发能力和自创动漫游戏品牌的创作主体，重点发展缤果动漫创作中心及周边产品研发中心等项目。

　　二、增强自主创新能力

　　把大力构建自主创新体系，作为推动新兴产业大发展、传统产业大转型的重要抓手，年内形成1—2个产业转型升级典型。

　　健全支持企业自主创新的机制。今年各级财政科技投入29.6亿元，比上年增长25.47%，通过加大财政投入引导全社会科技资金投入，推动企业成为科技投入和技术创新的主体。加大创新人才培养和引进力度，重点培养和引进战略性新兴产业发展的中高级技术人才、领军人才和创新科技团队。积极组织企业参与省部产学研战略联盟，全市组织实施产学研合作项目60项以上，推广科技成果500项以上。组织实施省市级科技计划项目300项以上。新培育扶持50 家规模以上企业创建高新技术企业或民营科技企业。

　　推进公共技术服务平台建设。完善国家级摩托车检测中心建设，加快国家半导体光电产品检测重点实验室、机械装备重点实验室建设，进一步推进制浆造纸、电声产品等省级检测站建设并争取成为国家级检测中心。鼓励企业和高校建立工程技术研发中心和申报组建省和国家重点实验室，培育创建企业工程研发中心10家以上。加强科技企业孵化器建设。大力推进专业镇技术创新试点工程。

　　大力实施质量强市战略。强化标准化工作，力争今年企事业单位参与制（修）订国际、国家、行业标准达10项以上。进一步推进名牌带动战略，力争新增5个省级以上名牌、5个省级以上名标。全市制造业信息化示范企业达到10家以上，传统产业企业信息技术应用率达到60%以上。加大知识产权保护力度，推进全市软件正版化工作。

　　三、加大基础设施和宜居城乡建设力度

　　加快推进交通能源基础设施建设。继续抓好广珠铁路江门段、佛开高速扩建、江番及江珠北延线高速、江罗高速、325国道复线I工程（含鹤南西江大桥）等重大交通基础设施项目建设，加快推进中开高速、新台高速南延线、广佛江珠城轨及江恩城轨（含台山支线）、沿海高铁的前期工作，以BT模式加快推进江顺大桥、西环路快速干道建设和金瓯路、江睦路、建设路立交等项目改造扩建。配合做好与港珠澳大桥的规划衔接工作，加快推进广海湾港区的规划建设，争取崖门3万吨级航道整治等项目提前动工。大力推进台山核电、台山电厂二期、新会双水发电厂热电联供机组、台山沿海及川岛风电等能源项目建设。

　　全力推进滨江新区和城乡建设。加快滨江新区13项重点基础设施建设进度，在抓好滨江体育中心立项设计、启动建设工作的同时，确保其余12项重点基础设施年内基本完成。推进主体功能区建设。进一步完善江门市及各县级市城市总体规划，优化城乡建设空间布局。加快推进“三旧”改造，继续完善省立绿道网功能配套，开展市域绿道与社区绿道规划建设，深入开展国家级生态乡镇、生态示范村、绿色社区、环境友好企业等创建工作，集中力量打造一批名镇名村，一年出成效，两年实现目标。

　　加大生态建设和环境保护力度。严格执行节能问责制和“一票否决”制。严格实行主要污染物排放总量控制制度，强力推进淘汰落后产能。抓好白沙电镀城搬迁等重污染行业定点建设工作。推广节能技术产品和合同能源管理模式，推进工业、交通、建筑、商贸酒店、公共机构等重点领域节能，抓好半导体照明产品示范工程。坚持实行总量前置审批和环境影响评价制度，禁止“两高一资”项目上马。全面推进规划环评，完善规划环评与项目环评的联动机制，促进重点产业合理布局。全面推进各级污水处理设施和配套管网建设，力争年内城镇污水处理率达72%。加强饮用水源保护区管理，加快推进乡镇饮用水源保护区划分与调整工作。积极推进循环经济、清洁生产和资源综合利用工作，建设低碳城市。

　　四、培育新的经济增长点

　　积极扩大城乡居民消费需求。严格执行并逐步提高最低工资标准，推行企业工资集体协商制度，确保各种强农惠农政策落实到农民头上，切实提高城乡居民收入，增加居民消费预期。全面实施国民旅游休闲计划，继续做好家电下乡、以旧换新工作。发展教育培训、家政服务、文化娱乐、体育健身等新型消费。加强农村市场网络建设。发展和完善消费信贷。着力保持物价稳定，大力实施“菜篮子”工程，扶持蔬菜大棚、冷藏设施和平价门店建设，切实稳定农副产品价格。完善价格异常波动应急机制和应急预案，增加对低收入群体的救助。尽快建立最低生活保障、失业保险标准与物价上涨挂钩的联动机制。

　　大力开拓国内外市场。推动企业巩固和深度发展美国、欧盟、香港、日本等传统市场，积极开拓东盟、南美、非洲、中东、俄罗斯、印度等新兴市场。支持企业走出去设立营销中心，进行跨国品牌推广。继续实施“科技兴贸”和出口品牌带动战略，促进“大通关”建设，优化出口商品结构。用好支持服务贸易出口的政策措施，扩大软件、技术、文化服务出口，培育我市知名服务贸易品牌。鼓励外向型企业创建内销品牌，推动外向型企业扩大内销。完善贸易摩擦应对防范和贸易救济机制。

　　增强民营经济发展活力和竞争力。制订优质企业培育成长计划，实施民营企业梯度培育工程和企业家素质提升工程，挑选一批发展潜力大、成长性好的民营中小企业，集中资源进行重点扶持。出台鼓励和引导民间投资指导目录，编制民间投资重点项目计划，积极扶持民营企业上市，扩大直接融资比例。鼓励民营企业创立具有自主知识产权的知名品牌。提高民营企业为跨国公司提供产业配套的能力，促进民营企业走集团化、国际化道路。健全面向中小企业的服务体系，搭建一批融资扶持、技术支持、创业辅导、人才培训、市场开拓等领域的公共服务平台。支持民营企业产品和服务进入政府采购目录。

　　五、加大城乡统筹力度

　　搞活农村经济。今年全市各级财政预算用于“三农”支出43.3亿元，比上年增长22.86%。要进一步完善财政支农资金管理体制，对重点农业项目的投入资金进行整合，提高资金使用效率。充分利用国家级现代农业示范区和省级粤台农业试验区两个平台，积极做好农业招商特别是对台招商工作。大力推广实用农业新技术，增加农业科技投入。加强农业机械化建设，进一步推广各类新型实用农机具。加强农业信息网络建设，进一步完善“农村信息直通车”等农业信息服务平台。继续深化农村综合改革，推进农村土地承包经营权有序流转和农村集体土地所有权确权工作，推进农村集体经济制度化、规范化管理和公司化改革。创新发展农村金融，进一步推进政策性农业保险。

　　构建现代农业体系。抓好我市农业产业发展规划的编制，引导农业园区建设和农业结构调整，优化农业产业布局，推进优势农业产业壮大。大力扶持和发展农业龙头企业、农民专业合作组织、农业经营大户等各类适应现代农业发展要求的经营主体扩大生产规模，建立农产品生产基地和农业园区。加强农产品流通服务体系建设，扶持壮大一批农产品物流企业发展。加大对优势特色农产品的培育力度，积极扶持农业名牌创建。加快海洋渔业发展，推进崖门国家一级渔港和广海国家二级渔港建设，把台山鳗鱼产业基地打造成国家级水产品出口基地。

　　推进城镇化建设。加快电信网、计算机网和有线电视网“三网融合”。继续完善中心镇基础设施建设，增强中心镇人口和产业集聚能力。完善城镇落户政策，帮助符合条件的农村富余劳动力向城镇转移就业落户。加快城市基础设施向农村地区延伸和覆盖。推进农村社区建设，加强镇一级城镇建设与管理机构建设，不断提高农村地区管理水平。

　　改善农村生产生活条件。积极探索建立全征地农民养老保障制度。抓好社会主义新农村建设，重点创建一批示范村，鼓励修建集中式农民公寓，实现农村面貌有明显改观，农民生活质量和文明素质有明显提高。加强以水利为重点的农村基础设施建设。通过产业扶贫、小额贷款扶贫、农村灾害保险等措施，抓好“双到”扶贫工作，力争三年任务两年完成。

　　六、进一步深化改革和推进一体化

　　深化重点领域和关键环节改革。积极稳妥推进大部制、富县强镇、简政强镇、事业单位分类等改革，进一步深化行政审批制度改革。理顺高新区管理体制，推进高新区“二次创业”。抓好鹤山作为省社会管理创新试点工作。以财力与事权相匹配为原则，推进全市性财政体制改革。深化户籍制度改革，做好农民工积分制入户城镇工作。继续做好集体林权制度改革的各项配套改革工作。认真做好申报全省海洋综合开发试点城市工作。

　　推进珠中江经济圈建设和珠三角五个一体化有关工作落实。按照实施《珠三角规划纲要》“四年大发展”的要求，以推进“五个一体化”为重点，全面落实《推进珠中江区域紧密合作框架协议》，力争我市跻身珠三角转变经济发展方式创新工作城市。推进珠中江公共交通“一卡通”。加快推进珠三角区域年票互认和高速公路联网收费。办好第二届珠中江进出口商品展销会。探索建立珠中江三地人力资源市场信息共享机制。进一步完善江门无线城市规划建设方案，促进珠中江信息通信资源的整合共享。加快推进市区自来水、清洁卫生、生活垃圾、有线电视、公交等价格收费标准的同城一体化。

　　七、加强以改善民生为重点的社会建设

　　今年各级财政预算民生投入61.4亿元，比上年增长25.08%，重点抓好八个方面的民生实事好事，让市民更多地享受到改革发展带来的实惠。

　　一是创建全国文明城市。今年是创建全国文明城市的冲刺之年，要全力打赢创文这一场硬仗，把创文作为一项提高城市管理水平和老百姓幸福指数的民生工程来抓。坚持创建为民，实现创建工作的制度化、常态化，广泛发动全民共建，强化突出问题的整改，确保硬件设施如期达标，通过创文进一步完善城市功能、规范城市管理、优化城市环境、提升市民素质。

　　二是促进就业。今年城镇新增就业4万人。继续深化“千企扶千村”就业工程，转移农村劳动力就业2万人。建立市创业孵化基地，创建创业带动就业平台。全面启动“和谐劳动关系示范区”创建工作。

　　三是扩大社会保障覆盖面。今年全市各级财政投入1.5亿元，实现新农保全覆盖。全面完善社会保险市级统筹。全市各级财政投入1亿元继续推进住房保障体系建设，新增廉租住房、公共租赁住房和经济适用住房等保障性住房1000套以上。将单亲特困母亲危房改造优先纳入住房保障范围。加快社区养老服务体系建设。提高低收入家庭生活保障，全市最低生活保障标准提高20元以上。落实孤儿最低养育标准。

　　四是抓好文化教育。今年全市各级财政教育投入27.76亿元，比上年增长18.74%，启动教育现代化建设工程，全面推动教育优质、公平和均衡化发展。强化提升中小学、幼儿园教师素质水平。大力发展学前教育特别是农村学前教育，全市100%镇（街）办有中心幼儿园。义务教育阶段规范化学校比例达到95%。整合优化市区高中阶段教育资源，全市普通高中优质学位达到100%，普通高中招生指标直接分配到初中学校的比例达到40%，建成广东省示范性中等职业学校1所。推行农民工子女积分制入读公办学校的有效机制，确保100%解决外来人口子女接受义务教育问题。推进高等教育向产学研纵深发展和更好地为地方服务。积极创建全国公共文化示范区。继续实施文化惠民工程，开展文化下乡进社区活动300场、东湖广场文艺演出52场，保证农村地区每个行政村每月放映一场电影，新建农家书屋190家。推进动漫影视产业园、江门演艺中心、市迎宾广场、星光公园二期等文化项目和设施建设。承办好中央电视台“中华情?江门月”中秋晚会。

　　五是保障市民健康。抓好医疗卫生工作，全面深化医药卫生体制改革。加强三级医疗卫生服务体系建设，促进城乡卫生服务均等化，让老百姓花更少的费用享受更便捷、更优质的医疗卫生服务。创新和完善公立医院运行机制，落实基本药物制度。完成12间乡镇卫生院中药房规范化建设。进一步做好农村创卫工作，新建或改建农村卫生户厕1万户。全面推进健康城市建设。加强重大疾病防控。推进城乡体育协调加快发展，普及全民健身设施建设，创建1个省级或全国体育先进社区，建设1个1.3万平方米的全民健身广场，广泛开展群众性体育活动。提高竞技体育水平，办好第二届全省农民运动会。

　　六是整治环境卫生。推进社会管理重心下移，逐步推广政府向社会购买服务。通过加大财政资金投入引导社会资金投入超1亿元，完成市区43个市场升级改造工作。完成天沙河改造。提高城市保洁水平，加强城乡生活垃圾收集、清运和无害化处理，大力整治“乱摆卖”、“牛皮癣”等城市痼疾和机动车尾气、油烟废气的超标排放。进一步绿化美化农村环境，以县（市）为单位每年整治10%的自然村。

　　七是促进交通畅通。进一步推行公交优先，今年市区更换和新增公交车220辆。年内完成东华大桥、白沙大道东、五邑路（东海路至胜利南路）扩宽工程建设，加快胜利大桥和胜利南路建设。完成农村公路建设120公里。启动轨道交通枢纽周边地段规划研究。加快落实公交站场的规划，辟建公交专用道，探索完善公共停车场的建设、管理机制。

　　八是建设平安江门。推进平安和谐社区建设，今年全市“六好”平安和谐社区要达80%以上。启动市直治安监控系统二期建设，新安装80个治安视频监控摄像头、12个治安视频卡口。建立安全生产长效机制，严格落实安全生产监管责任和企业安全生产主体责任，坚决遏制较大以上事故发生。加强市场监管，依法严厉打击制假售假、走私贩私、传销等违法活动。建立健全覆盖城乡的公共突发事件应急处置体系，提高政府有效应对自然灾害、事故灾难以及公共卫生、食品药品安全、社会安全、环境安全事件能力和群众自救互救能力。抓好信访维稳工作，落实领导“一岗双责”和包案责任制，发挥镇（街）综治信访维稳中心作用，支持检察机关派驻镇（街）检察室建设，切实提高社会矛盾纠纷排查化解工作能力。巩固民主法治村（居）创建成果。完善社会治安防控体系，加大“科技强警”力度，依法打击各类违法犯罪活动。

　　此外，要确保完成省下达的人口计生任务，人口自然增长率控制在6％。以内。维护妇女儿童合法权益。发展残疾人事业。加强国防教育，完善国防动员体系，落实“双拥”和优抚安置工作。继续做好民族、宗教、侨务、外事、港澳、对台、老龄、人防、统计、审计、档案、方志、气象、红十字会等工作。

　　八、抓好政府自身建设

　　创新政府服务。必须要以改革创新的精神，加快转变政府职能，分类整合行政资源，进一步提高为基层、为企业、为市民服务的水平。健全行政复议、投诉申诉、网络问政等制度，畅通民意表达渠道，完善“12345”政府服务热线办理机制，扩大公众对政府工作的有序参与。加强电子政务建设，深入推进信息资源整合共享，拓展网上受理、并联审批和一站式服务。

　　提升行政执行力。建立重大项目、重大活动和为群众办实事好事倒逼机制，以时限倒逼进度，以目标倒逼责任，分解目标，明确责任，把工作压力传送到每个环节和每个岗位，激发干部干事创业的干劲，狠抓各项工作的落实。主动接受人大监督，积极支持人民政协履行政治协商、民主监督、参政议政职能，扎实办理人大代表议案、建议和政协委员提案。配合人大开展好人大代表约见市长、局长活动。广泛听取各民主党派、工商联、无党派人士意见，支持工会、共青团和妇联等人民团体更好地发挥作用。坚持经济形势分析联席会议制度、经济社会发展顾问咨询制度、重大事项公开征集意见和合法性审查制度。树立高效务实的工作导向，强化绩效考核，加强效能监察、行政问责。

　　提高廉政建设水平。严格落实党风廉政建设责任制，进一步从源头上防治腐败。深化政务公开，完善电子监察系统并扩大监督范围，加强对政府性资金和投资项目的审计监督。严格财政预算编制执行，严控一般性支出，加强“三公”费用监管，严格执行党政机关厉行节约各项规定。进一步完善农村党风廉政信息平台。继续加大案件查处力度，严惩腐败分子，以反腐倡廉的实际成效取信于民。

　　各位代表，承继“十一五”辉煌，开创“十二五”伟业，是时代赋予我们的光荣使命。让我们在省委、省政府和市委的正确领导下，深入贯彻落实科学发展观，齐心协力，开拓进取，扎实工作，努力推动全市经济社会又好又快发展，以优异的成绩迎接中国共产党成立九十周年！
